# Macrophage Migration Inhibitory Factor Inhibition Is Deleterious for High-Fat Diet-Induced Cardiac Dysfunction

**DOI:** 10.1371/journal.pone.0058718

**Published:** 2013-03-11

**Authors:** Aurore Palud, Camille Marciniak, David Montaigne, Xavier Marechal, Caroline Ballot, Sidi Mohamed Hassoun, Brigitte Decoster, Remi Neviere, Steve Lancel

**Affiliations:** EA4484, Physiology Department, Lille 2 University, Lille, France; Pennington Biomedical Research Center, United States of America

## Abstract

**Aims:**

Development of metabolic syndrome is associated with impaired cardiac performance, mitochondrial dysfunction and pro-inflammatory cytokine increase, such as the macrophage migration inhibitory factor MIF. Depending on conditions, MIF may exert both beneficial and deleterious effects on the myocardium. Therefore, we tested whether pharmacological inhibition of MIF prevented or worsened metabolic syndrome-induced myocardial dysfunction.

**Methods and Results:**

C57BL/6J mice were fed for ten weeks with 60% fat-enriched diet (HFD) or normal diet (ND). MIF inhibition was obtained by injecting mice twice a week with ISO-1, for three consecutive weeks. Then, triglycerides, cholesterol, fat mass, glucose intolerance, insulin resistance, *ex vivo* cardiac contractility, animal energetic substrate utilization assessed by indirect calorimetry and mitochondrial respiration and biogenesis were evaluated. HFD led to fat mass increase, dyslipidemia, glucose intolerance and insulin resistance. ISO-1 did not alter these parameters. However, MIF inhibition was responsible for HFD-induced cardiac dysfunction worsening. Mouse capacity to increase oxygen consumption in response to exercise was reduced in HFD compared to ND, and further diminished in ISO-1-treated HFD group. Mitochondrial respiration was reduced in HFD mice, treated or not with ISO-1. Compared to ND, mitochondrial biogenesis signaling was upregulated in the HFD as demonstrated by mitochondrial DNA amount and PGC-1α expression. However, this increase in biogenesis was blocked by ISO-1 treatment.

**Conclusion:**

MIF inhibition achieved by ISO-1 was responsible for a reduction in HFD-induced mitochondrial biogenesis signaling that could explain majored cardiac dysfunction observed in HFD mice treated with MIF inhibitor.

## Introduction

A cluster of metabolic disorders that predisposes to cardiovascular diseases characterizes metabolic syndrome (MetS) [1]. Its prevalence is constantly rising and already ∼25% Americans and ∼15% Europeans are affected by MetS [2]–[4]. Patients with MetS have abdominal obesity in combination with two other metabolic abnormalities such as hypertriglyceridemia, low HDL cholesterol levels, raised blood pressure, glucose intolerance or type 2 diabetes mellitus [1]. Additional factors such as genetics, hormonal changes, and a proinflammatory state also contribute to the development of MetS.

Among the proinflammatory cytokines, several clinical studies established that macrophage migration inhibitory factor (MIF) levels positively correlated to body mass index, plasma free fatty acids and impaired glucose tolerance [5], [6]. In addition, elevated circulating MIF levels conferred to women, not to men, higher risks to develop type 2 diabetes and cardiovascular pathologies [7]–[9]. Thus, it is believed that MIF may be a key player in the development of cardiovascular disease associated with MetS although the underlying mechanisms are not fully understood.

MIF is a homotrimeric protein containing tautomerase activity [10]. It has been identified as a product of monocytes/macrophages [11], adipocytes [12], β pancreatic cells [13] or cardiomyocytes [14]. This pleiotropic cytokine participates to pathogenesis of inflammatory diseases including atherosclerosis [15], arthritis [15] and sepsis [16]. MIF contributes to regulation of glucose metabolism and is involved in development of insulin resistance [17]. MIF also directly targets cardiovascular system. Indeed, while MIF depresses myocardial function in a context of systemic inflammation [14], it exerts protective effects on the heart in circumstances of ischemia-reperfusion injury [18]. This beneficial impact of MIF has been attributed to changes in cardiac metabolism, i.e. enhanced cardiac glucose uptake [18].

As MIF may contribute to the pathogenesis of MetS-associated cardiomyopathy and modify cardiac metabolism, we tested the effects of MIF inhibition in mice fed with a fat-enriched diet. First, we sought whether induction of MetS in mice changed cardiac MIF expression along with myocardial dysfunction. Then, we tested the effects of pharmacological inhibition of MIF with ISO-1, a competitive inhibitor of tautomerase activity, on MetS-induced myocardial dysfunction. Finally, we determined whether MIF inhibition could modulate myocardial function through changes in cardiac glucose utilization and mitochondrial function.

## Materials and Methods

### Ethics Statement

All experiments were carried out in accordance with our national guidelines and approved by the “direction départementale de la cohésion sociale et de la protection des populations – Nord - Pas-de-Calais – Lille” (Permit Number 59-350206).

### Animals

Female C57BL/6J mice at 4 weeks of age were obtained from Charles River Laboratory (L’Arbresle, France). Mice had free access to tap water and were fed with either a standard chow diet (normal diet ND) or a high-fat diet (HFD, D12492 SSNIFF, Soest, Germany) in which 60% calories were from lard fat. Animals were kept under12 h light/dark cycles.

### ISO-1 Treatment

MIF inhibition was achieved by ISO-1 (Merck Chemicals, Darmstadt, Germany), a pharmacological compound binding to the tautomerase active site of MIF [19] and that has been widely used in various *in vivo* and *in vitro* experimental models [20]. After ten weeks of feeding, mice received ISO-1 injections [21] (20 mg.kg^−1^ total body weight, intraperitoneally) twice a week or the equivalent amount of DMSO used as solvent. After three weeks of treatment, mice were subjected to cervical dislocation. Fat mass from sub-cutaneous and abdominal regions was weighed and other parameters were measured.

### Plasma Analyses

Mice were fasted for six hours. Then, after tail incision, 100 µL of blood was withdrawn and collected into EDTA-coated capillary tubes. Plasma was obtained after a centrifugation performed at 1,000 *g*, at 4°C, for 15 min. Samples were aliquoted and stored at −80°C. Plasma true triglycerides were quantified by the use of the triglyceride determination kit from Sigma Aldrich (St Quentin Fallavier, France). Total cholesterol was measured with the cholesterol RTU kit, according manufacturer’s instructions (Biomérieux, Marcy-L’Etoile, France).

### MIF Activity

MIF tautomerase activity assay was performed as previously described [22]. Briefly, 1 µL of plasma was added in 499 µL of assay buffer (1 mM EDTA and 10 mM NaH_2_PO_4_, pH 6.4), and kept at room temperature for 2 min. Then, 500 µL of freshly prepared substrate solution (assay buffer containing 0.6 mM L-dihydroxyphenylalanine methyl ester and 20 mM Na m-periodate) were added into the cuvette, mixed and read at 475 nm using a spectrophotometer (Biomate 5″, Thermo electron corporation, Brebières, France). Decrease in optical density of the solution, corresponding to the consumption of L-dopachrome-methyl ester, was followed over 5 minutes. A positive control was achieved by adding 10 ng of mouse recombinant MIF protein (R&D Systems, Lille, France).

### Oral Glucose Tolerance Test (OGTT)

In order to assess glucose tolerance, animals were fasted for six hours. Then, mice received by gavage a glucose solution (2 mg.g^−1^ total body weight). Tail blood samples were taken before glucose administration and 10, 20, 30, 60 and 120 min after glucose ingestion. Glucose levels were measured using a glucose meter (Nova biomedical, Waltham, MA).

### Insulin Tolerance Test (ITT)

Insulin resistance was tested by injecting fasted mice intraperitoneally with insulin (0.5 mUI.g^−1^ total body weight). Blood glucose concentration was measured before injection and 15, 30 and 60 min later.

### Indirect Calorimetry

Mice were placed into a hermetic chamber connected to an indirect calorimetry system (Oxymax, Columbus Instruments, Columbus, OH). Animals had free access to tap water and food. Oxygen consumption (*V*O_2_) and CO_2_ production (*V*CO_2_) were recorded over 3 consecutive days. Respiratory quotient (RQ) was calculated as *V*CO_2_/*V*O_2_ ratio. Relative cumulative frequency curve analysis was used to determine substrate flexibility, as previously described [23], [24].

In another set of experiments, mice were subjected to an exercise stress test, as reported elsewhere [25]. Briefly, mice were placed on a treadmill enclosed into a metabolic chamber that was connected to the Oxymax apparatus. After a 30 min stabilization period, mice started running at 10 m.min^−1^ on a 0% incline and speed was incremented by 4 m.min^−1^ every 3 min until exhaustion. Exhaustion was reached when mice remained on the electrical shocker plate for 5 sec. Basal *V*O_2_ and maximal *V*O_2_ (*V*O_2_max) obtained during exercise were measured, allowing the calculation of Δ*V*O_2_ as *V*O_2_max-*V*O_2_.

### Myocardial Function

Freshly excised mouse hearts were immediately mounted onto a Langendorff apparatus, perfused at constant coronary flow (2.5 mL.min^−1^) with Krebs-Henseleit bicarbonate buffer and paced at 10 Hz. A latex balloon, inserted into the left ventricle, was filled with an aqueous solution to achieve a left ventricular end-diastolic pressure of 6–8 mmHg. The balloon was linked to a pressure transducer connected to ML118 bridge amplifier that fed into a Powerlab 8 SP high-performance data acquisition system (ADInstruments Ltd. by Phymep, Paris, France). Isovolumic contraction was appreciated by measuring left ventricular developed pressure (LVDP) and its first derivatives. Data were obtained with rising concentrations of the β-adrenergic receptor agonist isoproterenol (0, 1, 10 and 100 nM). Vasoreactivity was evaluated by perfusing sodium nitroprusside at different concentrations (0, 2, 20 and 200 µM).

### Mitochondrial Function

Freshly excised heart was rinsed, minced and homogenized into a mitochondrial extraction buffer (in mM: sucrose 300, TES 5, EGTA 0.2, pH 7.2) as previously described [26]. After centrifugations, mitochondrial pellets were suspended into mitochondrial respiration media Mitomed2 [27]. Mitochondria (50 µg) were placed into oxygraph chambers (O2K, Oroboros Instruments, Innsbruck, Austria) filled with 2 mL of Mitomed2, at 25°C. L-glutamate (10 mM) and L-malate (2 mM) were added in the first chamber, palmitoyl-L-carnitine (5 µM) and L-malate (2 mM) were added into the second chamber. After signal stabilization, ADP (2.5 mM) was injected and oxygen consumption was measured. Ratio substrates+ADP/substrates without ADP was calculated as a surrogate of mitochondrial coupling. At the end of the experiment, exogenous cytochrome c was added in order to test mitochondrial membrane intactness.

### Fructose-2,6-biphosphate Content Measurement

Fructose-2,6-biphosphate (Fru-2,6-P_2_) content has been evaluated as previously described by Hue et al. [28]. Briefly, flash frozen tissue was homogenized in 50 mM NaOH, heat up to 80°C for 10 min and cooled down. A solution containing acetic acid 25 mM and HEPES 20 mM was added until pH reached 8.0. After centrifugation at 15,000 *g* for 15 min at 4°C, supernatant was collected to measure its Fru-2,6-P_2_ content. Samples were added in a buffer containing: Tris 200 mM, pH 8.0, MgCl_2_ 100 mM, NADH 0.15 mM, aldolase 10 U, triose phosphate isomerase 10 U, glycerol-3-phosphate deshydrogenase (GDH) 10 U, phosphofructokinase : PPi, fructose-6-phosphate 5 mM. Reaction was initiated by adding 0.5 mM pyrophosphate. GDH-dependent NADH oxidation was followed by measuring absorbance at 340 nm for 5 min.

### Western-blotting

Whole cardiac tissue extracts were prepared as described previously [25]. Briefly, flash frozen hearts were homogenized in RIPA buffer (in mM: Tris 10; NaCl 140; EDTA 5; Triton X-100 1%; deoxycholate 1%; SDS 0.1%, pH 7.4; with antiprotease cocktail and antiphosphatases Na_3_VO_4_ 1 mM; NaF 20 mM) using a glass tissue grinder. After centrifugation, supernatants were collected and stored at −80°C until use. Proteins were separated by SDS-PAGE, transferred onto a nitrocellulose membrane and incubated with the following antibodies: anti-MIF (AbCam, Cambridge, UK), anti-GAPDH (Cell Signaling, Beverly, MA, USA). Blots were developed with ECL Plus reagent (GE Healthcare, Templemars, France). Protein concentration was determined using the Bradford assay.

### DNA and RNA Extractions

Total DNAs were extracted and purified by the use of QIAamp DNA Mini kit (Qiagen, Courtaboeuf, France), as stated by the manufacturer. Briefly, hearts were homogenized in the supplied tissue lysis buffer containing proteinase K. Then, samples were treated with RNAse A (Life Technologies SAS, Saint Aubin, France) and placed onto Qiagen Mini Spin columns. After washes, elution of DNA was achieved by adding 100 µL of AE buffer.

After tissue homogenization in Trizol reagent (Life Technologies SAS), total RNAs were purified with the PureLink Micro-to-Midi total RNA purification kit (Life Technologies SAS) according to manufacturer’s instructions. cDNA were prepared using transcriptor first strand cDNA synthesis kit (Roche Applied Science, Meylan, France).

### Real-time PCR and RT-PCR

Real-time PCRs and RT-PCRs were performed using Eppendorf Realplex S2 (Eppendorf, Le Pecq, France) and Mesa Blue qPCR Master Mix Plus for SYBR assay (Eurogentec, Angers, France). PCR primers used for determination of mitochondrial DNA copy numbers were *Ppia* (nuclear-encoded gene), *mtCOII* (mtDNA-encoded gene) and *Nd1* (mtDNA-encoded gene) and are presented Table S1. Primers used for RT-qPCR were against *Mif*, *Slc2a1*, *Pfkp*, *Pdk3* and *Pgc-1α* (Table S2). *β-actin* was used as an internal control (Table S2). Realplex software was used to quantify differences in gene expression. Results are expressed as fold expression.

### Statistical Analysis

Data are presented as means ± S.E.M. Statistical analysis was carried out using two-tailed unpaired t-test when comparing two groups and ANOVA when comparing three groups or more. Analysis was performed on GraphPad Prism software 5.0. Differences were considered significant when p<0.05.

## Results

### HFD Induced MIF Expression Increase

**Figure 1:**
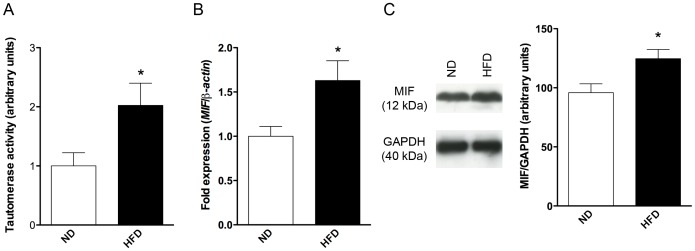
HFD induced systemic and cardiac MIF expression. (A) Tautomerase activity measured on plasma sample prepared from ND and HFD mice. Consumption of L-dopachrome-methyl ester was followed by measuring optical density at 475 nm. (B) Cardiac *Mif* transcript expression detected by RT-qPCR normalized to *β-actin*. (C) MIF protein cardiac expression observed by western-blot. Left panel is a representative western-blot, right panel is a semi-quantitation calculated after densitometric analysis. Data are means ± SEM. n = 6–10; *p<0.05 vs. ND.

First, we evaluated plasma tautomerase activity, which is mainly related to circulating MIF concentration. As shown in Figure 1A, tautomerization rate of L-dopachrome methyl ester into the dextrogyre isomer was doubled in plasma of HFD fed mice. As MIF is secreted by the heart to act as an autocrine/paracrine cytokine, we measured both *Mif* mRNA (Figure 1B) and protein (Figure 1C) in cardiac tissue. HFD induced elevation in cardiac MIF synthesis at both levels (Figures 1B and 1C).

### MIF Inhibition Worsened HFD-induced Cardiac Dysfunction

**Figure 2:**
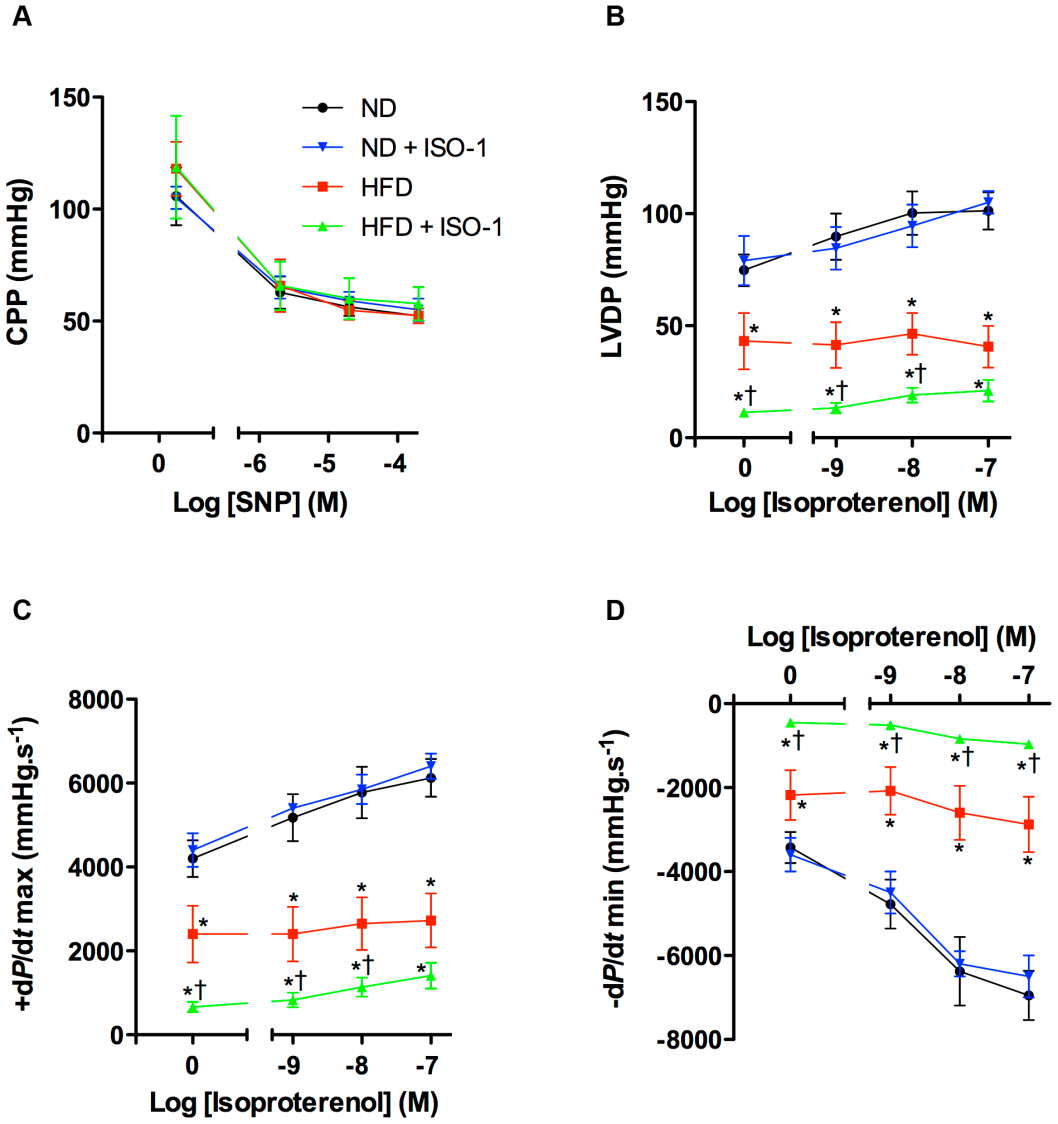
ISO-1 amplified HFD-induced cardiac dysfunction. (A) Coronary perfusion pressure (CPP) measured in presence of rising concentration of sodium nitroprusside (SNP) on isolated heart preparations obtained from ND, ISO-1-injected ND mice (ND+ISO-1), HFD and ISO-1-treated HFD mice (HFD+ISO-1). (B) Left ventricular developed pressure (LVDP), (C) +d*P*/d*t* max and (D) −d*P*/d*t* min measured on ND, ND+ISO-1, HFD, HFD+ISO-1 hearts, in presence of the β-adrenergic receptor agonist isoproterenol. Data are means ± SEM of 5 experiments. *p<0.05 vs. ND; ^†^p<0.05 vs. HFD.

Next, we tested the effects of the pharmacological MIF inhibitor ISO-1 on isolated hearts. Changes in LVDP were observed with no differences in coronary perfusion pressure (Figure 2A). LVDP (Figure 2B) as well as its first derivatives ±d*P*/d*t* (Figures 2C and 2D) were reduced in the HFD compared to ND mice. Addition of isoproterenol in the perfusion buffer exerted marked positive inotropic effects on hearts isolated from ND mice but not on hearts obtained from HFD mice (Figures 2B–2D). MIF inhibitor dramatically worsened HFD-caused cardiac dysfunction even in presence of the β-adrenergic receptor agonist (Figures 2B–2D). ISO-1 injection in ND mice did not alter LVDP (Figure 2B) and its first derivatives (Figures 2C–2D).

### MIF Inhibition had no Effects on Lipid and Carbohydrate Utilization

**Figure 3:**
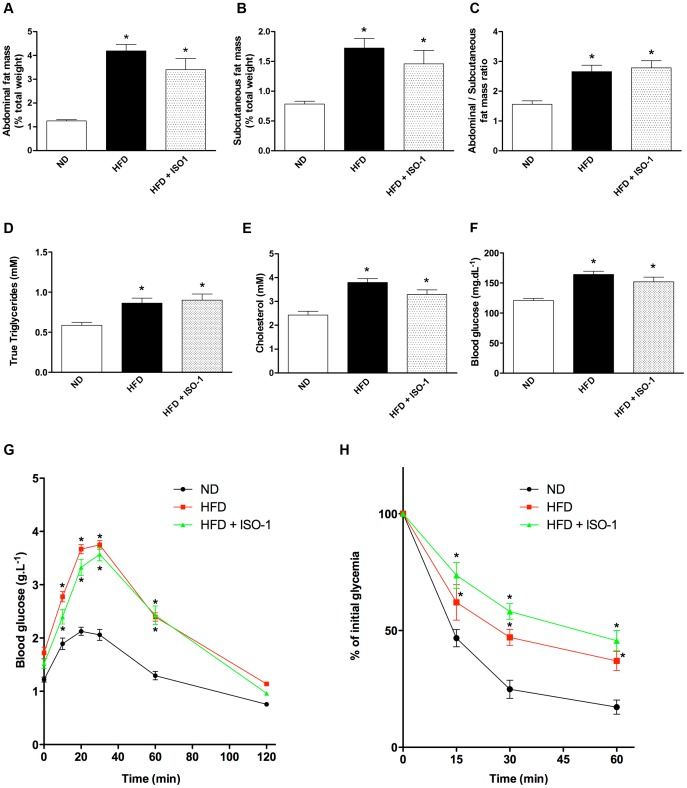
ISO-1 treatment did not modify metabolic syndrome-associated parameters. (A) Abdominal fat mass, (B) subcutaneous fat mass, (C) abdominal to subcutaneous fat mass ratio, (D) plasma true triglyceride levels, (E) plasma cholesterol concentration and (F) fasting blood glucose. Data are means ± SEM, n = 5; *p<0.05 vs. ND. (G) Oral glucose tolerance test and (H) insulin tolerance test performed on ND, HFD and HFD+ISO-1 mice. n = 5–10 in each group. *p<0.05 vs. ND.

We evaluated whether ISO-1 was responsible for an aggravation of metabolic parameters triggered by the high-fat regimen. HFD induced massive adipose tissue accumulation in the abdomen (Figure 3A) and in a lower extent in the subcutaneous compartment (Figure 3B), resulting in an elevation of the abdominal/subcutaneous fat mass ratio (Figure 3C). ISO-1 had no effects on fat mass amount and distribution (Figures 3A–3C). Similarly, both HFD and ISO-1-treated HFD mice had significant elevation of plasma true triglycerides and cholesterol compared to ND mice (Figures 3 D and 3E). In addition, ISO-1 did not reverse elevation in fasting blood glucose found in HFD mice (Figure 3F). Compared to ND mice, HFD or ISO-1-injected HFD mice were both intolerant to glucose and resistant to insulin, as suggested by the oral glucose tolerance (Figure 3G) and the insulin tolerance (Figure 3H) tests, respectively.

### MIF Inhibition did not Change HFD-induced Preferential Lipid Utilization as Substrate

**Figure 4:**
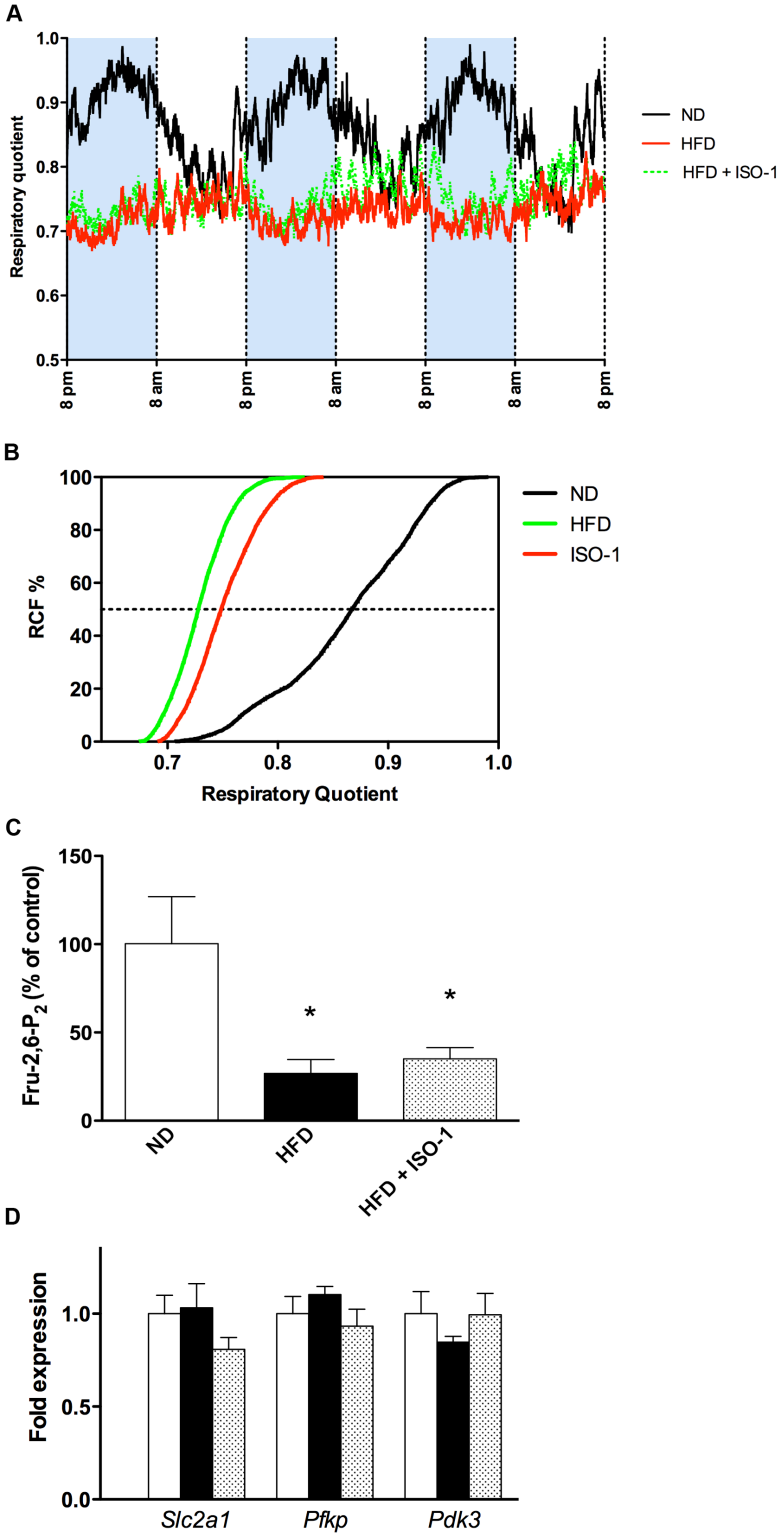
ISO-1 did not change animal energy balance and cardiac glucose utilization pathway. (A) Respiratory quotient, calculated as *V*CO_2_/*V*O_2_ ratio, over three consecutive days. Shaded areas between 8 pm and 8 am indicate dark periods. (B) Relative cumulative frequency (RCF) of respiratory quotient distribution. Experiments were performed on three mice in each group. Presented data are means only. (C) Fructose-2,6-biphosphate (Fru-2,6-P_2_) measured in cardiac lysates. (D) Cardiac expression levels of *Slc2a1*, *Pfkp* and *Pdk3* mRNAs. n = 4–5; *p<0.05 vs. ND.

Indirect calorimetry revealed that ND mice preferentially burned lipids during the light period and switched to glucose consumption during the dark phase, as suggested by the RQ oscillating from 0.7 to 1, respectively (Figure 4A). Thus, ND mice displayed broad substrate flexibility (Figure 4B). On the contrary, RQ of HFD mice remained close to 0.7 during light and dark periods, indicative of preferential lipid utilization (Figure 4A). HFD mice lost the phasic substrate flexibility observed in the ND mice (Figure 4B). ISO-1 injected in HFD mice did not restore substrate flexibility (Figures 4A and 4B).

These physiological integrated data supported preference for lipid utilization at the glucose expense in HFD mice. To determine whether such changes may occur in heart, we measured Fru-2,6-P_2_ in cardiac tissue. Fru-2,6-P_2_ is a co-activator of phosphofructokinase 1, an enzyme that stimulates glycolysis. Here, compared to ND, the amount of Fru-2,6-P_2_ was divided by three in the HFD and HFD+ISO-1 groups (Figure 4C), reinforcing the fact that glucose oxidation rate in the heart of these animals may be affected. Expression of genes encoding proteins involved in glucose metabolism was similar in all groups (Figure 4D).

### Indirect Calorimetry Revealed that MIF Inhibition was Associated with Changes in Oxygen Consumption

**Figure 5:**
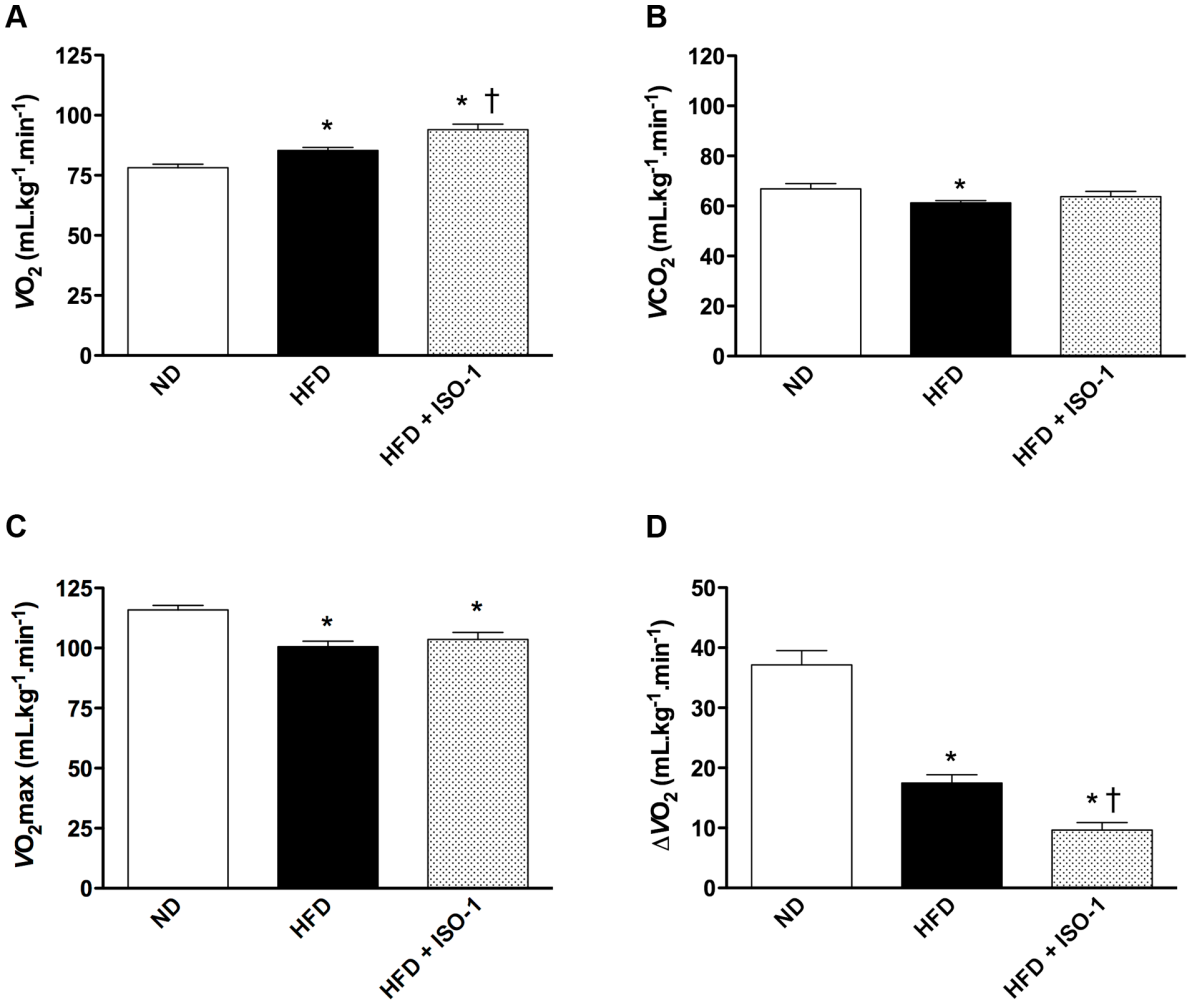
ISO-1 reduced aerobic capacity. (A) Basal *V*O_2_ and (B) *V*CO_2_ measured by indirect calorimetry. (C) Maximal *V*O_2_ (*V*O_2_max) obtained from mice subjected to treadmill exercise. (D) Difference between *V*O_2_ and *V*O_2_max (Δ*V*O_2_) in ND, HFD and ISO-1-treated HFD mice. n = 6–10, *p<0.05 vs. ND, ^†^p<0.05 vs. HFD.

Before exercise, compared to ND mice, HFD animals had higher oxygen consumption *V*O_2_ (Figure 5A) and a reduced CO_2_ production (Figure 5B). HFD mice treated with ISO-1 consumed even more oxygen than HFD mice (Figure 5A). These results were consistent with RQ observed during 24 hour-cycles (Figure 4A).

We next subjected mice to maximal physical exercise to test their maximal aerobic capacity as an indication of global mitochondrial function. When subjected to the treadmill test, elevation of *V*O_2_ was about 48% in the ND group while it increased by only 21% in the HFD group (Figure 5C), resulting in a significant reduction in Δ*V*O_2_ between ND and HFD mice (Figure 5D). At the exercise peak, *V*O_2_max was not significantly different between HFD and HFD+ISO-1 mice (Figure 5C). As a consequence, ISO-1-treated HFD mice had diminished Δ*V*O_2_ as compared to HFD mice (Figure 5D).

### MIF Inhibition did not Improve Mitochondrial Function but Reduced Biogenesis

**Figure 6:**
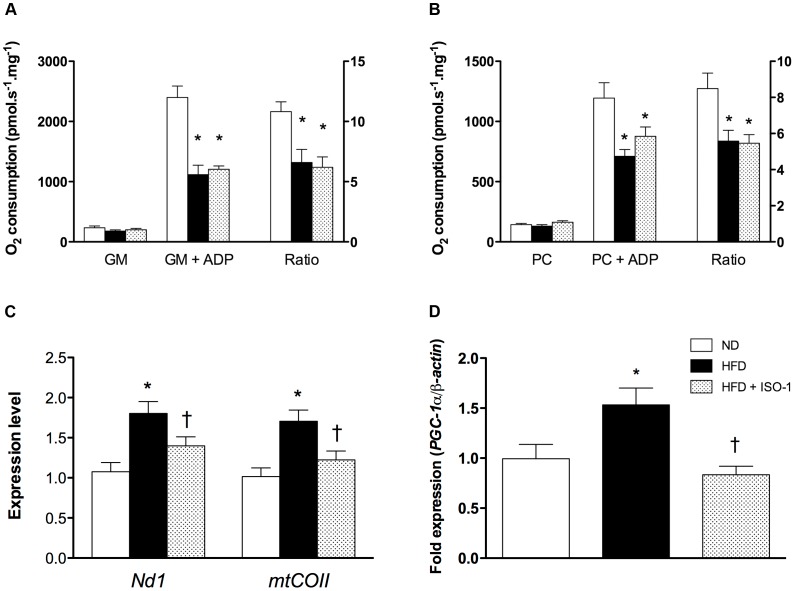
ISO-1 did not worsen mitochondrial function but blocked HFD-induced mitochondrial biogenesis. (A) Glutamate/malate (GM) and (B) palmitoylcarnitine/malate (PC) were used as substrates. Then, 2.5 mM ADP were added. Ratio was calculated as described in materials and methods. n = 6–10, *p<0.05 vs. ND. (C) Relative expression of mitochondrial-encoded genes *mtCOII* and *Nd1* compared to the nuclear-encoded gene *Ppia*. (D) Expression of *Pgc-1α* transcripts analyzed by real-time RT-PCR. n = 5, *p<0.05 vs. ND, ^†^p<0.05 vs. HFD.

We explored oxidative phosphorylation on isolated cardiac mitochondria with either glutamate/malate or palmitoylcarnitine/malate as substrates for respiratory chain complexes. In both conditions, in absence of ADP, no differences were observed between groups (Figures 6A and 6B). However, ADP-driven respiration was almost halved in HFD and HFD+ISO-1 compared to ND mitochondria (Figures 6A and 6B).

Mitochondrial biogenesis was evaluated by quantitative PCR targeting *mtCOII* and *Nd1*, two mitochondrial-encoded genes, and by measuring *Pgc-1α* transcript expression. Mitochondrial DNA copy number was significantly higher in the HFD group compared to ND mice as *mtCOII* and *Nd1* were increased (Figure 6C). On the contrary, *Nd1* and *mtCOII* in HFD+ISO-1 group were notably reduced compared to HFD (Figure 6C). Similar results were obtained for *Pgc-1α* expression (Figure 6D).

## Discussion

The causative link between MIF secretion and metabolic disorders has still to be demonstrated. Here, we induced metabolic disorders in mice that resembles to human MetS by the use of high-fat regimen. We also found that, in female mice, circulating level of MIF was increased, probably due to a massive release by adipocytes [12]. Such variations have similarly been reported in male animals [29]. Interestingly, we detected that heart increased its endogenous production of MIF. While this autocrine/endocrine function has been previously illustrated in sepsis [14] and ischemia/reperfusion injury [18], the overall cardiac effects of MIF are challenging as both protective and deleterious consequences have been reported [18], [30]–[32].

To test whether MIF participates to the HFD-induced cardiac dysfunction, we treated mice with ISO-1, an isoxazoline. This compound binds to MIF active site, hence inhibiting MIF tautomerase activity, which is required for part of its biological activity [33]. Although such a pharmacological approach may also have ‘off-target’ effects on MIF-sensitive metabolic organs such as liver, skeletal muscles or adipocytes [17], we observed that ISO-1 had no effects on cardiac function in ND mice but deteriorated contractile parameters in HFD animals. Consistent results have been recently reported in a type I diabetes mellitus model. For example, Tong et al. [34] found that cardiomyocytes isolated from streptozotocin-treated MIF knockout mice exhibited exacerbated contractile dysfunction compared to streptozotocin-treated wild-type mice. Thus, increased MIF production induced by HFD may be adaptive and exert cardioprotective effects, either directly or indirectly.

The importance of contractile dysfunction of heart isolated from ISO-1-treated HFD animals was striking because mice were still able to support sustained exercise running test. This may show some limitations of the Langendorff evaluation that does not take into account parameters influencing hemodynamic performance *in vivo* such as neuroendocrine regulation and circulating substrates that can be consumed by the heart. To explain marked deleterious effects of MIF inhibition on cardiac function, we measured metabolic parameters such as circulating triglycerides, cholesterol or insulin that may affect the myocardium energetics and contractile function. Contrary to other reports [35], [36], we did not detect any MIF inhibition-induced improvement of fasting blood glucose, glucose intolerance, insulin resistance or dyslipidemia. Of note, it should be pointed out that we injected ISO-1 when mice already had features of MetS [25] while most studies using pharmacological inhibitors [37], blocking antibodies [37] or genetically engineered mice [35] were performed in a prophylactic manner.

As measured circulating parameters were not affected by ISO-1, cardiac MIF production in response to HFD may represent a protective and adaptive response to maintain cardiac function. This contention is supported by the findings of Miller et al. that attributed beneficial effects of MIF in the ischemia/reperfusion injured heart to activation of the cardioprotective AMPK pathway and increased glucose uptake [18]. In the present study, endogenous cardiac energetic substrate utilization did not seem to be affected by MIF inhibition as the amount of Fru-2,6-P_2_ or genes involved in glycolysis remained similar in both HFD and HFD+ISO-1 animals. Consistently, indirect calorimetry revealed that both HFD and HFD+ISO-1 groups had a similar RQ, at around 0.7, meaning that a higher proportion of fat is oxidized at the expense of carbohydrates. Beside availability of heart fuels, cardiac energy production also relies on mitochondria number and efficiency. As previously reported [25], cardiac mitochondrial respiration from HFD mice was reduced either in presence of Krebs cycle or β-oxidation -related substrates. ISO-1 did not deteriorate respiration of isolated mitochondria, suggesting that intrinsic mitochondrial function was similar in both HFD groups. However, indirect calorimetry experiments revealed that basal oxygen consumption (in the amount of which the heart is likely the principal oxygen-consuming organ) was increased in HFD+ISO-1 mice compared to HFD mice. Taken together, these results prompted us to study cardiac mitochondrial mass, whereas we could not rule out changes in other oxygen-consuming tissues such as skeletal muscles. Specifically, we studied mitochondrial population by measuring mtDNA copy number and *Pgc-1α* expression as mitochondrial biogenesis hallmarks [38]. Interestingly, ISO-1 abrogated HFD-induced increases in mtDNA copy number and *Pgc-1α* expression. Thus, although the understanding of underlying molecular mechanisms warrants further studies, cardiac MIF expression in response to HFD may somehow participate to mitochondrial biogenesis and improve energy production.

Beside the mitochondrial hypothesis, other mechanisms, including calcium mishandling and reactive oxygen species (ROS) production, may explain why ISO-1 was deleterious for HFD hearts. Calcium mishandling is a well-known feature of diabetes-related cardiomyopathy [39]. For instance, reduced sarcoplasmic endoplasmic reticulum calcium ATPase (SERCA) activity has been described in different models [40], [41]. In the present study, isoproterenol failed to increase cardiac force in both HFD and HFD+ISO-1 mice, suggesting disturbances in calcium homeostasis. Thus, regarding the dramatic reductions in cardiac force, contractility and relaxation parameters (± d*P*/d*t*) observed in the HFD+ISO-1 group, it can be hypothesized that MIF inhibition had worsened calcium properties in cardiac cells. However, opposite data on the role of MIF on calcium homeostasis has been published. On the first hand, calcium amplitude currents through L-type channels are reduced in presence of recombinant MIF in atrial cells [42]. On the other hand, further altered calcium transients have been observed in adult cardiomyocytes isolated from streptozotocin-treated MIF knockout mice compared to streptozotocin-treated wild-type mice [34]. Eventually, apart from its tautomerase activity, MIF bears antioxidant properties. Typically, Koga *et al.* [30] reported that MIF was able to reduce oxidative stress in the post-ischemic heart. In our model of HFD, massive amount of circulating lipids may lead to myocardial oxidative stress due to lipotoxicity. Thus, HFD-induced cardiac MIF expression may represent a protective response towards increased oxidative stress. Nevertheless, a recent study showed that MIF itself was able to induce ROS production and subsequently autophagy in hepatocarcinoma cells [43].

In conclusion, MIF inhibition by ISO-1 worsened HFD-induced cardiac dysfunction without any effects on metabolic parameters. However, ISO-1 injections in HFD mice led to a reduced mitochondrial biogenesis signaling that may, in turn, lead to an insufficient ATP supply required for cardiac contraction.

## Supporting Information

Table S1 **Primer sequences used for qPCR.**
(DOCX)

Table S2 **Primer sequences for RT-qPCR.**
(DOCX)
