# Supplementary material for: Macrophage Migration Inhibitory Factor Inhibition Is Deleterious for High-Fat Diet-Induced Cardiac Dysfunction
Source: PLoS One. 2013 Mar 11;8(3):e58718. doi: 10.1371/journal.pone.0058718 (PMC3594150; doi:10.1371/journal.pone.0058718)
Supplement: Table S1 — Primer sequences used for qPCR. (DOCX) [file pone.0058718.s001.docx]

**Table S1: Primer sequences used for qPCR.**

| Gene | Gene name | Primer sequences |
| --- | --- | --- |
| *Nd1*  *(mitochondrial)* | *NADH dehydrogenase subunit 1* | F: GTTGGTCCATACGGCATTTT  R: TGGGTGTGGTATTGGTAGGG |
| *mtCOII*  *(mitochondrial)* | *Cytochrome c oxidase subunit II* | F: ACGAAATCAACAACCCCGTA  R: GGCAGAACGACTCGGTTATC |
| *Ppia*  *(genomic)* | *Peptidylprolyl isomerase A* | F: ACACGCCATAATGGCACTGG  R: CAGTCTTGGCAGTGCAGAT |
