# Supplementary material for: Macrophage Migration Inhibitory Factor Inhibition Is Deleterious for High-Fat Diet-Induced Cardiac Dysfunction
Source: PLoS One. 2013 Mar 11;8(3):e58718. doi: 10.1371/journal.pone.0058718 (PMC3594150; doi:10.1371/journal.pone.0058718)
Supplement: Table S2 — Primer sequences for RT-qPCR. (DOCX) [file pone.0058718.s002.docx]

**Table S2: Primer sequences for RT-qPCR.**

| Gene | Gene name | Primer sequences |
| --- | --- | --- |
| *Mif* | *Macrophage migration inhibitory factor* | F: CCATGCCTATGTTCATCGTG  R: AGGCCACACAGCAGCTTACT |
| *Slc2a1* | *Solute carrier family 2 (facilitated glucose transporter), member 1* | F: AACACTGGTGTCATCAACGC  R: GAGTGTGGTGGATGGGATG |
| *Pfkp* | *Phosphofructokinase, platelet* | F: AGCTATCGGTGTCCTGACCA  R: TAAAGTACACTTTGGCCCCC |
| *Pdk3* | *Pyruvate dehydrogenase kinase, isoenzyme 3* | F: CGTCGCCACTGTCTATCAAA  R: GTTAGCCAGTCGCACAGGA |
| *Pgc-1α* | *Peroxisome proliferator-activated receptor, gamma, coactivator 1 alpha* | F: CGGAAATCATATCCAACCAG  R: TGAGGACCGCTAGCAAGTTTG |
| *Actb* | *Actin, beta* | F: AGCTGCCTGACGGCCAGGTC  R: GCTCAGGAGGAGCAATGATC |
